# Fine mapping of a thrips resistance QTL in *Capsicum* and the role of diterpene glycosides in the underlying mechanism

**DOI:** 10.1007/s00122-021-03790-6

**Published:** 2021-02-20

**Authors:** Pauline van Haperen, Roeland E. Voorrips, Martijn van Kaauwen, Henriëtte D. L. M. van Eekelen, Ric C. H. de Vos, Joop J. A. van Loon, Ben Vosman

**Affiliations:** 1 Plant Breeding, Wageningen University and Research, P.O. Box 386, 6700 AJ Wageningen, The Netherlands; 2 Laboratory of Entomology, Wageningen University and Research, P.O. Box 16, 6700 AA Wageningen, The Netherlands; 3 Present Address: Keygene N.V, P.O. Box 216, 6700 AE Wageningen, The Netherlands; 4 Bioscience, Wageningen University and Research, PO Box 16, 6700 AA Wageningen, The Netherlands

## Abstract

**Key message:**

A major thrips resistance QTL in *Capsicum* was fine-mapped to a region of 0.4 Mbp, and a multidisciplinary approach has been used to study putative underlying mechanisms.

**Abstract:**

Resistance to thrips is an important trait for pepper growers. These insects can cause extensive damage to fruits, flowers and leaves on field and greenhouse grown plants worldwide. Two independent studies in *Capsicum* identified diterpene glycosides as metabolites that are correlated with thrips resistance. In this study, we fine-mapped a previously defined thrips resistance QTL on chromosome 6, to a region of 0.4 Mbp harbouring 15 genes. Two of these 15 candidate genes showed differences in gene expression upon thrips induction, when comparing plants carrying the resistance allele in homozygous state to plants with the susceptibility allele in homozygous state for the QTL region. Three genes, including the two genes that showed difference in gene expression, contained a SNP that was predicted to lead to changes in protein structure. Therefore, these three genes, i.e. an acid phosphatase 1 (APS1), an organic cation/carnitine transporter 7 (OCT7) and an uncharacterized locus LOC107874801, are the most likely candidates for playing a role in thrips resistance and are a first step in elucidating the genetic basis of thrips resistance in *Capsicum*. In addition, we show that the diterpene glycoside profiles did not differ between plants with the resistance and susceptibility allele for the chromosome 6 QTL, suggesting that these compounds do not play a role in the resistance conferred by the genes located in the major thrips resistance QTL studied.

**Supplementary Information:**

The online version of this article (10.1007/s00122-021-03790-6).

## Introduction

Thrips are major pest insects in crops worldwide, both in the field and in the greenhouse (Kirk and Terry 2003; Morse and Hoddle 2006). Thrips can cause direct damage to the crops by feeding on fruits, flowers and leaves, leading to their deformation, reduced growth, altered carbon allocation, and thus reduced quality and yield (Welter et al. 1990; Shipp et al. 1998). Thrips can also damage plants indirectly by transmitting viruses such as tomato spotted wilt virus (TSWV) (reviewed by Jones (2005)). Current protective measures only partially affect thrips population growth, because of their high reproduction rate, short life cycle, thigmotactic behaviour, which hampers early detection, and their ability to feed on multiple host plants and to develop resistance to insecticides (Hansen et al. 2003; Bielza 2008). Biological control employing predation by *Orius* spp. (Hemiptera: Anthocoridae) can limit thrips population growth, but predator pressure should remain high, as thrips population growth will occur when the predator population is too small (Sanchez and Lacasa 2002; Tommasini et al. 2004). Therefore, natural host plant resistance against thrips is a highly desired trait.

Screening wild accessions for thrips resistance and subsequent quantitative trait loci (QTL) mapping of the resistance factor(s) are the first steps to identify candidate genes that play a role in thrips resistance and in developing thrips-resistant varieties. Previous studies have identified QTLs involved in resistance to *Thrips palmi* Karny in common bean (*Phaseolus vulgaris*) (Frei et al. 2005) and to *Megalurothrips sjostedti* Trybom (Omo-Ikerodah 2008; Sobda 2017), *Thrips tabaci* Lindeman and *Frankliniella schultzei* Trybom (Muchero et al 2010) in cowpea (*Vigna unguiculata*). However, in all these cases further fine mapping of the QTLs is still needed to identify the gene or genes that are involved in thrips resistance.

Other studies focussed on the potential role of secondary plant metabolites in thrips resistance. Leiss et al. (2009a) showed that concentrations of the pyrrolizidine alkaloids (PA) jacobine and jaconine and the flavonoid kaempferol glucoside were higher in resistant *Senecio* plants. In thrips-resistant chrysanthemum, high concentrations of chlorogenic acid (caffeoyl quinic acid) and feruloyl quinic acid were found (Leiss et al. 2009b). Moreover, an unsaturated isobutylamide was suggested to repel thrips in chrysanthemum (Tsao et al. 2005). In the leaves of thrips-resistant carrots, relatively high amounts of luteolin, sinapic acid and ß-alanine were found (Leiss et al. 2013). Mirnezhad et al. (2010) and Vosman et al. (2018) showed that high levels of acylsugars are associated with thrips resistance in tomato.

In pepper, Maharijaya et al. (2011) identified the wild *Capsicum annuum* accession CGN16975 as a potential source for thrips resistance. Further characterization of the resistance showed that the larval development is inhibited on young leaves (Maharijaya et al. 2012; Van Haperen et al. 2019), and that resistance was functional in several *Capsicum* backgrounds (Van Haperen et al. 2020). QTL mapping for resistance to *Frankliniella occidentalis* was carried out in an F_2_ population derived from an interspecific cross between the resistant *Capsicum annuum* CGN16975 and the susceptible *Capsicum chinense* CGN17219 (Maharijaya et al 2015). A single QTL that explained around 50% of the genetic variation was identified on chromosome 6. The resistance parameters used in this QTL analysis, i.e. larval survival from the L1 to L2 stage, larval survival from L2 to pupal stage, and leaf damage in a no-choice assay, all co-localize near the same marker on chromosome 6. No additional QTLs were found. The gene or genes that confer thrips resistance in *Capsicum* are unknown.

Maharijaya et al. (2019) combined QTL mapping and an untargeted metabolomic approach to detect metabolite QTLs (mQTLs). Six mQTLs, of which four metabolites were correlated with thrips resistance, co-located with the thrips resistance QTL on chromosome 6. These four metabolites included two acyclic diterpene glycosides and a flavonoid conjugate. In an independent study by Macel et al. (2019), monomer and dimer acyclic diterpene glycosides were identified as metabolites related to thrips resistance when comparing leaves of thrips-resistant and susceptible *Capsicum* accessions. Altogether, these two studies suggest a possible role of diterpene glycosides (capsianosides) in thrips resistance in *Capsicum*.

The aim of this study was to fine map the QTL on chromosome 6 in order to identify the gene or genes that may play a key role in thrips resistance in *Capsicum*, and to determine if diterpene glycosides can be linked to the resistance mechanism. We used plants that originated from the F_2_ mapping population that was developed by Maharijaya et al. (2015). In addition, we used gene expression data to determine which genes in the fine-mapped QTL region are most likely to play a role in thrips resistance. Subsequently, an untargeted metabolomic approach was used to determine whether or not diterpene glycosides, or other metabolites, are more abundant in F_4_ plants derived from the mapping population (Maharijaya et al. 2015) that contain the resistance allele for the QTL region in homozygous state, compared to plants from the same F_4_ line that have the susceptibility allele in homozygous state. In addition, we determined whether genes known to play a role in diterpene glycoside biosynthesis are located in the fine-mapped QTL.

## Material and methods

### Overview of experiments and plant material

The plant material used for QTL validation and fine mapping originated from the F_2_-mapping population of Maharijaya et al. (2015) (Table 1), which was based on the resistant accession *C. annuum* CGN16795 and the susceptible accession *C. chinense* CGN17219. Eleven F_2_ plants that were heterozygous for the 2-LOD QTL support interval were selfed. For validation of the QTL, five F_3_ lines obtained by selfing F_2_ plants heterozygous for the 2-LOD QTL support interval were selected (F_3_-lines 1–5, Table 1). For each line, larval development on plants with the resistance (“R”) allele in homozygous state was compared to larval development on plants with the susceptibility (“S”) allele in homozygous state.

**Table 1:** Plant lines used in validation and fine mapping of the QTL region on chromosome 6

Plant line	QTL chromosome 6	Experiment
CGN16975A	R	QTL validation and fine mapping
CGN17219A	S	QTL validation and fine mapping
F3 lines 1–5	R or S	QTL validation
F3 lines 1–10	Recombination	Fine mapping
F4 lines 1–9	Recombination, R or S in homozygous state	Fine mapping and validation
F5 lines 1–4	Recombination, R or S in homozygous state	Fine mapping and validation
F4 line 10	R or S	Gene expression and metabolomics

CGN16975A and CGN17219A were included as resistant and susceptible references. Plants with the resistance allele (“R”) or susceptibility allele (“S”) in homozygous state were selected to validate the QTL region on chromosome 6. F3 plants with a recombination in the QTL region were selected for phenotyping to fine map the resistance QTL. F3 plants with a recombination in the new fine-mapped region were then selfed for one or two generations, and F4 or F5 plants with the recombination in homozygous state were selected for phenotyping to further fine map the QTL and to validate the fine-mapped QTL region

For fine mapping of the resistance QTL, F_3_ plants with a recombination in the QTL region were selected. These F_3_ plants were phenotyped and subsequently selfed for one or two generations, in order to obtain F_4_ and F_5_ lines comprising plants with the recombination in the new fine-mapped region in homozygous state, or the unrecombined “R” or “S” allele in homozygous state, to further fine map and validate the new QTL region. Selfings from CGN16975 (CGN16975A; first generation) and CGN17219 (CGN17219A; second generation) were included as resistant and susceptible references, respectively. Seeds were sown in potting compost in a greenhouse of Unifarm (Wageningen University and Research, Wageningen, the Netherlands). Plants were grown with a photoperiod of L16:D8 and 70% RH at 25 °C. No insecticides were applied. Thrips were controlled using the *Orius laevigatus* (Fieber) (Entocare C.V., Wageningen, the Netherlands). Plants were watered three times a week. Nutrients were added two times a week.

Gene expression and metabolite composition and/or content were studied in two groups of plants, one of plants with the “R” allele and one of plants with the “S” allele in homozygous state. A heterozygous F_3_ plant for the fine-mapped QTL region on chromosome 6 (M11 to M15) was selfed to obtain F_4_ plants with the “R” or “S” allele in homozygous state (F_4_ line 10, Table 1; Table S1). For the metabolite analysis CGN16975A and CGN17219A were included as reference. Plants were sown and grown under the same conditions as described above.

### DNA extraction

Young leaves of each individual plant were collected for DNA extraction in tubes containing two 2-mm stainless steel beads each and stored in a -80 °C freezer until use. Samples were ground with a TissueLyser II (Qiagen) at 25 Hz for 70 s. Microprep buffer was prepared as described by Fulton et al. (1995), and 500 µL was added to each ground leaf sample. The samples were shaken until homogenized and incubated at 65 °C for 30 min, after which they were cooled for 5 min in ice water and 500 µL chloroform was added. After gently shaking the samples 40 times, the samples were centrifuged at 3500 rpm for 15 min. The supernatant was transferred to a new tube and 0.8 volume of isopropanol was added. The samples were inverted 40 times and centrifuged at 3500 rpm for 15 min. The supernatant was discarded. The pellet was washed with 175 µL 70% ethanol and dried. The pellet was re-suspended in 100 µL MQ and stored in the freezer at -20 °C until use.

### Molecular marker design

Single nucleotide polymorphism (SNP) discovery was carried out on RNAseq data of the F_1_ that resulted from a cross between the parents of the F_2_-mapping population. RNA extracted from the leaves of the F_1_ plant was sequenced by BGI Genomics. SOAPdenovo (Luo et al. 2012) was used to make the assembly. The reads were mapped to the assembly using Bowtie2 (Langmead and Salzberg 2012). We used qualitySNPng to detect SNPs (Tang et al. 2006; Nijveen et al. 2013). Fifteen SNP markers (Table S1) were selected in the previously identified QTL region (Maharijaya et al. 2015). Forward and reverse primers were designed using Primer3Plus (Untergasser et al. 2007).

An additional marker for fine mapping of the resistance was designed based on the whole genome re-sequencing of the resistant accession *Capsicum annuum* CGN16975 (M13.4). DNA was extracted from CGN16975A and sent to Novogene Technology Co., Ltd (Hong Kong) for library preparation and sequencing. Reads were mapped to the *Capsicum annuum* Zunla-1 assembly (Qin et al. 2014) and the UCD10X assembly (Hulse-Kemp et al. 2018) using BWA-mem (Li 2013). The Integrative Genomics Viewer (Robinson et al. 2011) was used to find SNPs and their flanking sequences. Primers were designed using Primer3Plus (Untergasser et al. 2007).

### Genotyping

Plants that were used to validate the previously identified QTL region and to fine map the thrips resistance were genotyped by Dr. van Haeringen Laboratorium B.V. (VHL Genetics company, Wageningen, Netherlands) with two SNP markers (M2 and M15, Table S1), flanking the 2-LOD interval of the QTL region as determined by Maharijaya et al. (2015), using KASP assays (Semagn et al. 2014). F_3_ plants with a recombination between these flanking markers were genotyped with additional markers M6, M7, M8, M10, M12, M13 and M14 in the QTL region using KASP assays by Bejo Zaden B.V., Warmenhuizen, the Netherlands (Table S1). Additional markers in the area of interest (M10.1, M10.2, M10.3, M10.4, M11 and Isotig 18,067–441; Table S1) were tested with LightScanner^®^ System (Idaho Technology Inc.) using the small amplicon approach (Liew et al. 2004). F_4_ and F_5_ plants that were used to further fine map and validate the new QTL region were genotyped using KASP assays for M10, M10.1, M10.2, M10.3, M10.4, M12, M13, M14 and M15, and LightScanner assays for M11 and M13.4. LightScanner assays for M11, Isotig 18,067–441 and M15 were used to genotype and select plants for differential gene expression and metabolomic analysis.

### Thrips rearing and synchronization

The population of *Frankliniella occidentalis* originated from Greenhouse Horticulture of Wageningen University and Research (Bleiswijk, the Netherlands). *Frankliniella occidentalis* was reared in a growth cabinet at 25 °C, L16:D8, 70% relative humidity, on *Phaseolus vulgaris* beans in glass jars covered with thrips-proof gauze. Synchronized first instar larvae (L1) were obtained by allowing female adult thrips to lay eggs on snack cucumbers. After 24 h, the female adults were brushed off and the cucumber was kept in the growth cabinet at 25 °C. Synchronized L1s emerged after 4 days.

### Detached leaf assay

Thrips resistance based on larval development was determined in a detached leaf assay, as previously described by Van Haperen et al. (2019). A high correlation between outcomes of tests quantifying resistance in whole plants and tests on detached leaves was previously shown (Maharijaya et al 2011), indicating that the resistance mechanism is functioning in detached leaves. We selected the youngest fully opened leaves of each plant. The detached leaf was placed with the petiole in a droplet of 1.5% water agar in a Petri dish (BD Falcon, tight-fit lid 50 × 9 mm). Five synchronized L1 larvae were placed on each leaf, after which the Petri dish was firmly closed and incubated in a growth cabinet at 25 °C. Thrips development was determined at 3, 5 and 7 days post infestation. The level of thrips resistance was determined by the fraction L1 that did not develop into second instar larvae (L2). Development of L1 into L2 occurs in 1–2 days under optimal conditions (Lublinkhof and Foster 1977). Three leaves per F_3_ plant (F_3_ line 1–5, Table 1) of 12 weeks old plants were used to validate the QTL region identified by Maharijaya et al. (2015). Four leaves of 12 weeks old F_3_ plants with a recombination were used to fine map this QTL. For a further fine mapping step, F_3_ plants with a recombination within the newly mapped QTL region were selfed for one or two generations, and three leaves per F_4_/F_5_ plant of 10 weeks old plants were used to further fine map and validate the new region.

To study the expression of genes in the QTL region upon induction with L1 larvae, the youngest fully opened leaves of 12 weeks old plants were collected and placed in Petri dishes as described above. Half of the leaves was infested with 15 synchronized L1s. After six hours, all leaves were frozen in liquid nitrogen and stored at − 80 °C until RNA extraction. This time point was selected as follows:(1) it was expected that the resistance mechanism is activated in an early stage of L1 to L2 development, and (2) several differentially expressed genes upon thrips infestation are still strongly expressed after 5 h (Sarde et al. 2019; Sarde 2019).

### RNA extraction and sequencing

Plants from the F_4_ line 10 with the resistance (“R”) or susceptibility (“S”) allele in homozygous state between M11 and M15 were selected for RNA sequencing in two different treatments (i.e. induced: infested with 15 L1 larvae, or mock-treated: not infested). Fifteen plants with genotype R and fifteen plants with genotype S were randomized in the greenhouse. Leaves of the plants were collected and either infested or mock-treated, as described in the previous paragraph. Three pools of leaves from five plants with the same QTL genotype (“R” or “S”) and treatment (induced or mock) were ground to a fine powder with mortar and pestle in liquid nitrogen. Total RNA was extracted from the pooled samples using RNAeasy Plus Mini Kit (QIAGEN) and sent to Novogene Technology Co., LTD (Hong Kong), for library preparation and sequencing. STAR (Dobin et al. 2012) was used to map the reads to the Zunla-1 assembly and the UCD10X assembly. Read count files were generated using HTSeq (Anders et al. 2014). The Genome Data Viewer of NCBI was used to list the candidate genes in the fine-mapped QTL region (https://www.ncbi.nlm.nih.gov/). The analysis of differentially expressed genes was done using SARTools in R-3.5.2 (R Core Team 2018; Varet et al. 2016). To analyse whether SNPs are predicted to lead to changes in protein structures, RNA sequences of each group (“R” and “S”) were translated into protein sequences using ExPASy (Gasteiger et al. 2003). SNPs that translated into different amino acids were analysed using PROVEAN tools to determine whether the change in amino acid is predicted to lead to a change in protein structure (Choi et al. 2012).

### Metabolite extraction and profiling using LC–MS

Every sample consisted of a pool of ten leaves, either infested or mock-treated, taken from five plants of the same QTL genotype, i.e. with either the “R” allele or the “S” allele in homozygous state, or from the resistant or susceptible reference. The leaves were ground into a fine powder in liquid nitrogen, and 300 mg fresh weight of leaf powder per sample was used to extract their metabolites. Two technical quality control samples (TC), consisting of mixed powder from different samples, were included. One of the pooled samples of group R, mock-treated, dropped out due to a technical problem of the LC–MS. Metabolites were extracted with 900 µL 99.87% MeOH containing 0.13% formic acid. LC–MS was performed using an HPLC system (Dionex Ultimate) coupled to an Q Exactive Orbitrap FTMS mass spectrometer (Thermo Scientific) using a Phenomenex Luna C18 column and a gradient of 5–75% acetonitrile in 0.1% formic acid in 45 min (Xu et al. 2019). Alternating positive–negative electrospray switching mode was used at a mass resolution of 60,000 (full width at half maximum) and a mass range of m/z 90–1350 D. Metalign software (Lommen 2009) was used to extract and align mass peaks in an unbiased manner. Mass peaks that were predicted to belong to the same metabolite were clustered by MSClust software (Tikunov et al. 2012). From here on, these mass peak clusters will be referred to as “metabolites”.

### Data analysis and statistics

Fractions L1 were transformed by *y* = arcsine(√x) before analysing the data. A Student’s t test was used to determine whether the transformed fractions L1 significantly differed between plants with the “R” or “S” allele within the same F_3_ line to validate the previously identified QTL region. Student’s t test was also used to determine significant differences in resistance between plants that had the recombination in homozygous state and plants with the “R” or “S” allele within each F_4_ or F_5_ line. All statistical analyses were conducted using Genstat 18th edition (VSN International 2015).

In the metabolite data, non-detects were randomized with values between 45,000 and 55,000 ion counts, i.e. 45–55% of the local noise as determined by the Metalign software. The data were transformed as *y* = log10(x), and t tests were used to generate P-values. False-discovery rate correction (FDR) with *α* = 0.10 was used to correct for multiple comparisons (Benjamini and Hochberg 1995). A principal components analysis (PCA) was carried out using SIMCA version 15.02 (Umetrics, Umea, Sweden). The PCA plot was based on the variation in metabolite profiles. Data were normalized across samples with Pareto scaling before carrying out the PCA analysis. As one of the three thrips-exposed samples from group S clearly deviated from the two other biological replicates, this sample was excluded from the analysis, as most likely this sample was taken from group R rather than group S.

The genome annotation *Capsicum annuum* Zunla-1 from the CNGBdb (https://db.cngb.org/search/assembly/GCF_000710875.1/; Accessed on January 31, 2021) was used to identify genes in the QTL region that might conceivably play a role in diterpene glycoside biosynthesis. The initial steps in the diterpene glycoside biosynthesis occur through the methylerythritol 4-phosphate (MEP) pathway in the plastids (Lange et al. 2000). Along the MEP pathway, the precursors of the terpenoids, i.e. isopentenyl diphosphate (IPP) and dimethylallyl diphosphate (DMAPP) are produced (Supplementary data Table S2A). Condensation of one DMAPP molecule and three IPP molecules yields geranylgeranyl pyrophosphate (GGPP), which is needed to synthesize diterpenes (Bohlmann et al. 1998; Takahashi and Koyama 2006). These precursors are the substrates of enzymes called terpene synthases (TPSs) for the formation of terpenes (Chen et al. 2011) (Supplementary data Table S2B). Other enzymes such as Cytochrome P450 monooxygenases and UDP-glycosyl transferases (UGTs) also play a role in terpenoid glycoside synthesis (Collu et al. 2001; Richman et al. 2005). BLAST was used to determine the physical position of two markers flanking the region where six mQTLs that co-localized with the resistance QTL were located on the Zunla-1 assembly (Qin et al. 2014; Maharijaya et al. 2019) (HmpsE088 and HmpsE113; Table S3).

## Results

### QTL validation

**Fig. 1:**
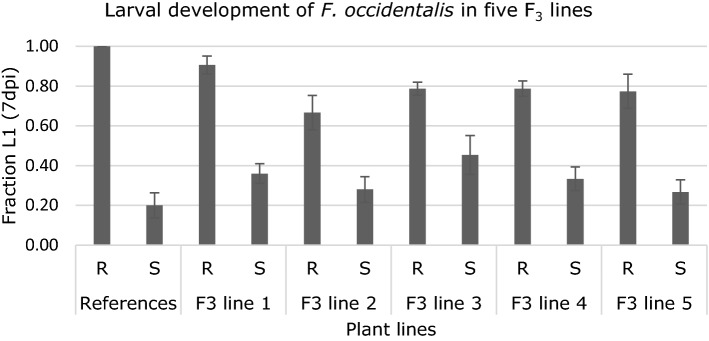
Validation of the QTL for thrips resistance in Capsicum. Larval development of F. occidentalis was studied on leaves of five F3 lines with either the resistance (“R”) or susceptibility (“S”) allele in homozygous state. CGN16975A and CGN17219A were included as resistant (“R”) and susceptible (“S”) references. All differences between plants from group “R” and group “S” within the same line were significant (*P* < 0.05)

To validate the previously identified QTL in the lines that were derived from the mapping population of Maharijaya et al. (2015), plants from five F_3_ lines that either have the resistance allele (“R”) or susceptibility allele (“S”) in homozygous state in the 2LOD interval region between M2 to M15 were scored for first instar larval development (Table S2). A high fraction L1 larvae indicates thrips resistance. As expected, plants with the “R” allele showed a significantly higher fraction L1 compared to plants with the “S” allele for all F_3_ lines, confirming the previously identified QTL (Fig. 1).

### Fine mapping and validation

Among the 2000 plants from F_3_ line 1–10, 120 F_3_ plants were identified with a (heterozygous) recombination between markers M2 and M15 that flanked the QTL region. In order to fine map the resistance locus, these 120 plants were phenotyped (Supplementary data Table S4). The strongest correlation between fraction L1 larvae and marker genotype (RR, RS, SS) in the QTL region was found for marker M12.

**Fig. 2:**
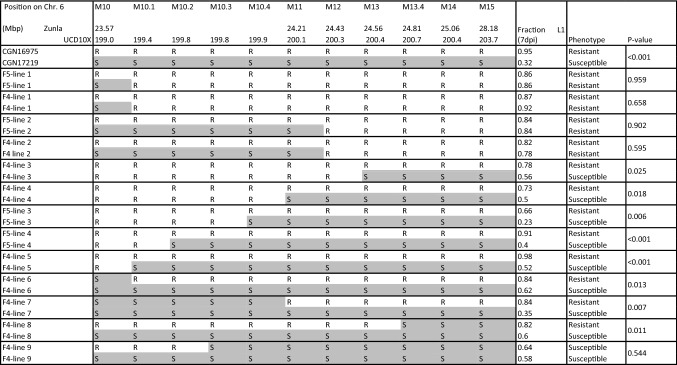
Validation of the fine mapping of the thrips resistance QTL on chromosome 6. For each marker (M10 to M15), “R” and “S” denote the presence of the resistant parent (CGN16975) or susceptible parent (CGN17219) allele in homozygous state, respectively. The levels of thrips resistance between groups of plants with contrasting QTL genotypes within the same line are considered significantly different when *P* < 0.05

In order to validate this result and to further narrow down the interval, 13 F_3_ plants with a recombination between M10 and M15 were selfed for one or two generation and the F_4_ and F_5_ plants with the recombination in homozygous state were selected. They were compared to plants from the same line with the “R” or “S” allele in homozygous state (Fig. 2). From the results of F_4_ line 3, it appears that the causal gene is located to the right of marker M12, while from the results of F_4_ line 8 we conclude that it is located to the left of marker M13.4. The physical distance between those markers is 0.39 Mbp according to the Zunla-1 assembly and 0.40 Mbp according to the UCD10X assembly.

### Candidate genes

Nine genes are predicted in the region between the flanking markers (M12 and M13.4) based on the *C. annuum* Zunla-1 assembly (Qin et al. 2014). In the UCD10X assembly of CM334, the region between M13.4 and M14 is inverted (Hulse-Kemp et al. 2018). Therefore, the six genes in the region between M13.4 and M14 are also included in the overview of candidate genes (Table 2).

**Table 2:** Predicted genes in the fine-mapped QTL region on chromosome 6

Location (Zunla-1, chromosome 6)	Gene symbol	Gene description
24.42–24.43 Mbp	LOC107876103	Acid phosphatase 1-like
24.46–24.47 Mbp	LOC107876104	Uncharacterized LOC107876104
24.52–24.52 Mbp	LOC107876105	Profilin-2
24.53–24.54 Mbp	LOC107874799	Putative protein TPRXL
24.54–24.55 Mbp	LOC107876106	Protein IQ-DOMAIN 31-like
24.56–24.56 Mbp	LOC107876107	Hexokinase-2
24.56–24.57 Mbp	LOC107876108	Nuclear poly(A) polymerase 4-like
24.73–24.74 Mbp	LOC107876109	Organic cation/carnitine transporter 7-like
24.81–24.82 Mbp	LOC107876110	Organic cation/carnitine transporter 7-like
24.83–24.84 Mbp	LOC107874800	Uncharacterized LOC107874800
24.95–24.95 Mbp	LOC107874801	Uncharacterized LOC107874801
24.96–24.96 Mbp	TRNAK-CUU	Transfer RNA lysine (anticodon CUU)
24.97–24.97 Mbp	LOC107873994	F-box/LRR-repeat protein At3g59250-like
25.01–25.01 Mbp	LOC107873995	Uncharacterized LOC107873995
25.06–25.07 Mbp	LOC107876111	Uncharacterized LOC107876111

The gene list is based on the nine predicted genes on the Capsicum annuum Zunla-1 assembly for the region between marker M12 and M13.4. The six predicted genes between M13.4 and M14 are also included. The Genome Data Viewer from NCBI is used to enlist the predicted genes and their gene description. Expression data of these predicted genes can be found in Supplementary data (Table S2)

**Fig. 3:**
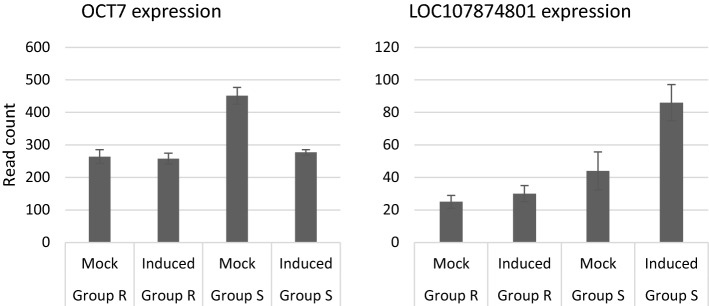
Differentially expressed genes in the fine-mapped QTL region on chromosome 6. Group “R” and “S” indicate the groups of plants that have the resistance (“R”) or susceptibility (“S”) allele in homozygous state for the QTL region. “Induced” indicates the leaves that were induced with 15 L1 larvae for six hours, whereas no L1s were added to the “Mock” treated leaves. OCT7 and LOC107874801 show significant differences in gene counts between “Mock” and “Induced” treatments within “Group S” (*P*-adjusted for multiple comparisons < 0.05)

After a significant difference in thrips resistance levels between F_4_ plants from group “R” and group “S”, i.e. with the resistance allele (“R”) or the susceptibility allele (“S”) in homozygous state, was confirmed (Supplementary data Fig. S1), the RNAseq data of these two groups of plants, in two different treatments, i.e. thrips-exposed or mock-treated, were used to compare differentially expressed genes (DEGs) in the fine-mapped QTL region. Two DEGs were found in the region of interest, i.e. LOC107876110, an organic cation transporter (OCT7) and LOC107874801, an uncharacterized locus (Fig. 3). We observed significantly lower counts of OCT7 reads in the “S” group when leaves were induced with L1 larvae compared to mock-treated leaves (Fig. 3, left panel). In the “R” group, we did not observe significant differences between mock and induced leaves. We observed a significant increase in counts of the uncharacterized locus (LOC107874801) reads upon induction in the “S” group (Fig. 3, right panel) and no significant differences between the “R” groups with different treatments. Expression of other genes in the QTL region did not significantly differ between the groups or treatments (Supplementary data Table S5).

Three genes in our region of interest have SNPs that are predicted to lead to changes in protein structures, based on the analysis with ExPASy and PROVEAN (Supplementary data Table S6A-C), including the two genes found in the DEG analysis (OCT 7 and LOC107874801); the third gene is an acid phosphatase 1 (APS1). The allele that has a mutation that was predicted to lead to an altered protein structure was observed in the susceptible group for all three genes.

### Metabolomics

Metabolites were extracted from leaves of F_4_ plants that either have the R-allele or S-allele in homozygous state between M11 and M15, and the resistant and susceptible references, both exposed to L1 larvae and mock-treated leaves. In negative ionization mode, which was the most sensitive mode for detecting diterpene glycosides, a total of 1363 putative metabolites were detected. Forty of these compounds were putatively identified as diterpene glycosides, as they contained a positive ionization mode fragment with a mass to charge ratio (m/z) of 271.2410, i.e. [C20H31]^+^, which is an indicative fragment of diterpene glycosides (Heiling et al. 2016). Thirty-nine of these compounds were present in all tested samples. One of these putative diterpene glycosides was only detectable in the resistant reference accession but not in the R group, in both thrips-exposed and mock-treated plants (Table 3).

**Table 3:** Detected compounds (mass cluster ID) that were putatively identified as diterpene glycosides, and their relative abundance in the CGN16975, CGN17219, group R and group S, either mock-treated or L1-exposed

Cluster ID	#peaks in cluster	Mass	Retention time (min)	CGN16975		CGN17219		Group R		Group S	
				Mock-treated	Thrips-exposed	Mock-treated	Thrips-exposed	Mock- treated	Thrips-exposed	Mock-treated	Thrips-exposed
1168	20	983.471558	23.59	6.09	6.28	6.46	6.65	7	7.26	7.1	6.88
1262	32	907.382141	24.86	6.43	6.37	5.87	5.81	6.88	6.94	6.95	6.98
1311	26	341.091125	25.6	7.28	7.25	5.86	5.9	5.91	6.28	6.13	6.09
1323	46	1185.519653	25.79	7.74	7.66	5.74	5.76	5.13	5.29	5.07	5.16
1328	31	999.459595	25.88	7.57	7.57	5.79	5.87	5.72	5.96	5.82	5.64
1342	16	659.328308	26.08	6.89	6.87	5.62	5.6	6	6.18	6.13	5.88
1378	63	1129.528809	26.66	8.74	8.75	6.41	6.56	6.21	6.72	6.09	5.96
1417	61	1169.523193	27.42	8.76	8.73	6.99	6.83	6.39	6.57	6.58	6.48
1491	42	967.475647	28.62	8.71	8.73	8.92	8.93	8.92	8.97	8.93	8.88
1517	86	497.275421	29.06	8.18	8.22	7.34	7.25	7	7.03	7.04	6.87
1543	11	1007.470764	29.43	8.68	8.65	9.01	9.01	9.01	9.02	9.02	9
1558	23	271.242035	29.62	6.43	6.53	6.13	6.24	6.64	6.84	6.68	6.45
1582	101	1021.951172	29.96	8.08	8.03	6.08	6.12	6.05	6.14	6.16	6.34
1592	25	1007.470764	30.12	8.01	7.99	8.76	8.76	8.77	8.8	8.8	8.77
1610	92	1709.854614	30.38	8.33	8.37	5.96	6.17	6.05	6.21	6.18	6.08
1628	48	271.242004	30.61	7.49	7.46	6.02	5.99	5.65	5.65	6.01	5.37
1668	19	271.242035	31.18	7.64	7.56	6.63	4.68	6.49	5.82	6.27	5.73
1723	81	940.925598	31.87	8.46	8.42	5.78	5.75	5.87	5.87	5.96	5.83
1751	46	1593.805298	32.29	8.21	8.25	8.1	8.18	8.32	8.38	8.3	8.17
1773	44	1589.738281	32.61	6.14	6.19	7.68	7.62	7.56	7.62	7.7	7.78
1785	19	1633.80127	32.82	8.36	8.34	8.43	8.46	8.51	8.52	8.51	8.49
1814	32	859.396484	33.33	8.48	8.48	8.67	8.68	8.78	8.8	8.83	8.78
1836	43	1447.748535	33.66	7.34	7.53	8.24	8.27	8.22	8.27	8.22	8.15
1857	54	859.396667	34	7.93	7.91	8.31	8.34	8.51	8.54	8.56	8.52
1863	10	271.242035	34.13	5.09	5.05	6.13	6.16	6.27	6.29	6.31	6.32
1875	19	867.393677	34.47	7.17	7.2	6.51	6.55	6.41	6.51	6.57	6.43
1890	42	1573.743896	34.71	7.28	7.32	8.6	8.57	8.54	8.56	8.58	8.62
1907	29	1447.75	35.1	7.42	7.64	8.22	8.25	8.18	8.25	8.2	8.11
1919	6	359.020813	35.33	6.47	6.29	4.66	4.66	5.36	4.66	5.15	4.95
1928	19	1487.744019	35.53	7.65	7.69	8.52	8.53	8.49	8.51	8.5	8.49
1948	49	1573.744629	35.93	7.54	7.55	8.62	8.59	8.54	8.56	8.6	8.62
1960	3	271.242035	36.14	5.32	5.39	Not detected	Not detected	Not detected	Not detected	Not detected	Not detected
1967	37	1487.744385	36.39	6.42	6.5	8	7.98	7.92	8	8	8
1981	44	1573.744629	36.85	6.43	6.45	8.22	8.17	8.06	8.11	8.15	8.23
2008	30	1573.743896	37.77	5.09	5.21	7.35	7.28	7.15	7.21	7.32	7.39
2042	12	1471.752563	38.86	4.42	4.31	7.02	6.94	6.39	6.43	6.55	6.55
2121	53	856.876892	41.58	6.11	6.12	6.18	6.2	7.79	7.99	7.73	7.54
2141	2	271.241821	42.33	4.7	4.83	5.2	4.7	4.7	4.7	4.7	4.7
2238	4	853.457153	46.08	4.7	4.7	4.7	4.7	4.7	4.7	4.7	4.7
2264	4	566.346436	47.02	6.45	6.45	5.99	6.01	5.64	5.76	5.7	5.6

The columns indicated by CGN16975, CGN17219, Group R and Group S indicate the log10 of the relative abundance of thrips-exposed and mock-treated samples

**Fig. 4:**
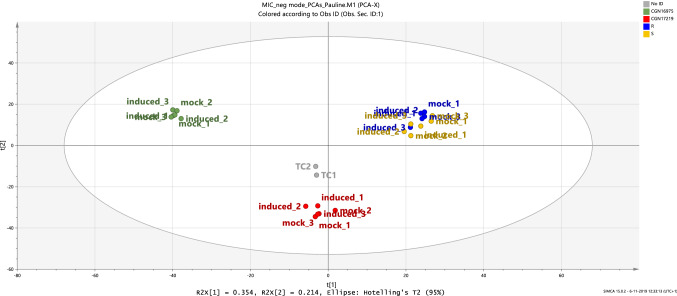
Principal components analysis (PCA) plot of the thrips-exposed and mock-treated Capsicum leaf samples based on their metabolite profiles determined by untargeted LCMS. Green dots correspond to the resistant (CGN16975A) reference samples, red dots to the susceptible (CGN17219A) reference samples, blue dots to the samples from group R and yellow dots to the samples from group S. TC1 and TC2 correspond to the technical quality control samples. Each sample is a pool of 10 leaves from 5 individual plants. The explained variance is 56.8%

**Fig. 5:**
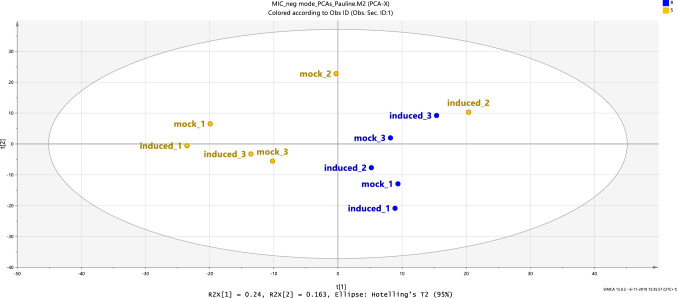
PCA plot of leaves of a subset of plants, i.e. from both group R and group S, either after exposure to first instar larvae or mock- treated, based on their metabolite profiles. Blue dots represent samples from group R, yellow dots samples from group S. The PCs explain 40.3% of the variance

The PCA plot based on the metabolite profiles of all samples, i.e. both group R, group S and the resistant (CGN16975A) and susceptible (CGN17219A) reference, thrips-exposed and mock-treated, shows a grouping of plants into 3 different clusters (Fig. 4). A clear separation between the resistant and susceptible references is observed, resulting in two distinct clusters of samples. The third cluster of samples contains the leaf extracts of both group R and group S F_4_ plants. We did not observe a clear separation between mock-treated and thrips-exposed samples, neither within the clusters for the resistant and susceptible references, nor within the group R and group S cluster (Fig. 5).

**Fig. 6:**
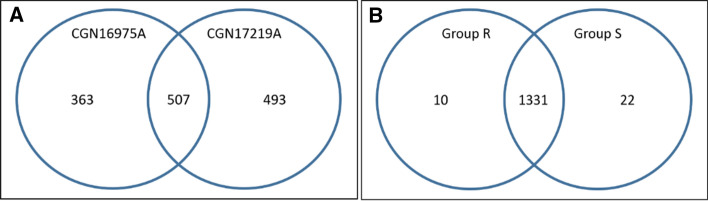
Overview of the number of differentially abundant metabolites between the resistant (CGN16975A) and susceptible (CGN17219A) references (panel A), and between group R and group S (panel B) in negative mode. Numbers indicate the number of metabolites that are significantly more abundant in relative intensity in one of the two plant groups (*P* adjusted < 0.05), or that did not significantly differ between these two groups (numbers in common between group circles). These numbers include some artefacts, including the 10 and 22 in panel B

After correcting for false-discovery rate due to multiple testing, 856 metabolites were found to differ significantly in their relative abundance between the resistant and susceptible references for both treatments (Fig. 6a; Supplementary data Table S7). Thirty-six of these metabolites could be annotated as diterpene glycosides, based on their specific fragment in positive ionization mode, of which 18 were more abundant in the resistant, and the other 18 more abundant in the susceptible reference accession. When considering the effect of thrips feeding, we found that within the susceptible reference two metabolites were significantly different between thrips-exposed and mock-treated samples, while one metabolite was significantly different within the resistant reference between thrips-exposed and mock-treated samples (Supplementary data Table S8). Between group R and S of the F_4_ lines, 32 metabolites differed significantly in their relative abundance for both treatments (Fig. 4b; Supplementary data Table S9). In group R, two metabolites differed significantly in relative abundance between the thrips-exposed and mock-treated groups (Supplementary data Table S10). In group S, four such metabolites were observed. However, all putative compounds that significantly differed between group R and group S, or between thrips-exposed and mock-treated within group R and S, respectively, are most likely artefacts. Such artefacts may result from false-positive eluent mass peaks picked up by the unbiased processing procedure, which can occur by, e.g. matrix-dependent local differences in ionization suppression of the LC eluent (Antignac et al. 2005). The same applies to metabolites that significantly differed between group R and group S, or in group R and group S between mock-treated and thrips-exposed samples picked up in the positive ionization mode. In addition, none of the detected compounds that was significantly different between thrips-exposed and mock-treated leaf samples of F_4_ plants contained the positive m/z 271.2410 fragment that is characteristic for diterpene glycosides.

### Diterpene biosynthesis genes within the resistance QTL

The flanking markers of the mQTLs that co-localize with the resistance QTL as defined by Maharijaya et al. (2015) (Table S3) were mapped to the Zunla-1 assembly at physical positions 5.77 Mbp and 43.14 Mbp (Maharijaya et al. 2019). Between these flanking markers, 812 genes were predicted according to the Pepper genome database. Twenty-one of these 812 genes putatively encode enzymes that may be involved in the diterpenoid biosynthesis pathway, of which 15 genes encode a cytochrome P450 (Supplementary Table S11) and six genes encode, respectively, a geranylgeranyl transferase subunit, three gibberellin 20-oxidases, and two gibberellin 3-beta-dioxygenases. Only one of these genes, Capana06g001248, putatively encoding cytochrome P450 94A2, is located in the fine-mapped QTL region (24.43–24.81Mbp).

## Discussion

### Validation and fine mapping of the resistance QTL

The evaluation of F_3_ plants that have the resistance or susceptibility allele in homozygous state in the previously described QTL region on chromosome 6 (Maharijaya et al. 2015) showed that plants with the resistance allele had a significantly higher fraction L1, indicating that larval development is impaired and thus confirming the role of the previously defined QTL region. This (partial) inhibition of larval development affects population development, as only part of the L1 larvae is able to develop into the next larval stage on the leaves expressing the resistance. This way, the resistance mechanism not only plays a role in controlling the insect population growth through interruption of the thrips life cycle, but also in the spread of tospoviruses. Thrips can only acquire tospoviruses such as TSWV in the L1 stage or early L2 stage, and re-infect plants through their saliva during the adult stage (Whitfield et al. 2005), thus interrupting the development of L1 larvae into next developmental stage is a promising mechanism to prevent or limit tospovirus outbreaks. It has been shown that the spread of TSWV in a thrips-resistant pepper accession is less severe and delayed compared to the spread in a thrips-susceptible pepper accession, most likely due to decreased population development in the thrips-resistant accession (Maris et al. 2003). Previous thrips resistance QTL mapping studies used reproductive adaptation (Frei et al. 2005), damage score (Frei et al. 2005; Omo-Ikerodah 2008; Muchero et al. 2010; Maharijaya et al. 2015; Sobda 2017) or larval and pupal development and survival (Maharijaya et al. 2015) as parameters for thrips resistance. By validating the QTL region using larval development as a resistance parameter, we confirm that this method is appropriate to further fine map the resistance gene(s).

Fine mapping of the resistance QTL resulted in narrowing down our region of interest to an interval between markers M12 and M13.4. The physical distance between these markers is 0.39 Mbp based on the Zunla-1 assembly and 0.40 Mbp based on the UCD10X assembly (Qin et al. 2014; Hulse-Kemp et al 2018). As we only found a few plants with a recombination between M12 and M13.4 in our screening of 2500 plants, thousands of plants would have to be screened for further fine mapping between these two markers. Therefore, we used gene expression and gene functionality to further limit the number of candidate genes in the current interval.

### Candidate genes

Fifteen candidate genes are predicted in the current fine-mapped QTL region on chromosome 6. Five of the predicted genes are uncharacterized loci. Based on the differential gene expression analysis and the prediction of SNPs that lead to protein structural changes, three genes, of which one is an uncharacterized locus, are the most likely candidates in the resistance mechanism.

The first candidate is an organic cation/carnitine transporter (OCT7), which plays a role in plant adaptation to environmental stresses such as cold, drought and salt stresses in *A. thaliana* (Küfner and Koch 2008). It can transport organic cations, nicotinate and compounds such as trigonelline (Berardini et al. 2015). Trigonelline has also been connected to thrips resistance. Mirnezhad et al. (2010) observed lower amounts of this compound in thrips-resistant tomato accessions. They hypothesized that this was a trade-off to favour acyl sugar production. The proteins predicted to result from the susceptibility and resistance alleles of OCT7 differ, with one amino acid change in the susceptible allele predicted to be deleterious. This might lead to non-functional or less than optimally functioning transporters, leading to disruptions of cation or carnitine balances and deficiencies. In *Arabidopsis*, a mutation in organic cation transporter 1 (AtOCT1) affected the expression of carnitine-related genes and led to developmental defects (Lelandais-Brière et al. 2007). Also, we observed that the level of expression of OCT7 in mock-treated plants that have the susceptibility allele in homozygous state was significantly higher than OCT7 expression in mock-treated plants with the resistance allele in homozygous state. The expression of OCT7 in the susceptible group of plants was decreased upon induction with L1 larvae. This might indicate that high expression of OCT7 in the susceptible group of plants initially favours larval development. Also, it seems that the difference between plants carrying the susceptibility or resistance allele can be explained by the mutation, rather than the expression levels of OCT7, as the expression levels of OCT7 do not significantly differ between the thrips-exposed plants with the susceptibility allele in homozygous state, compared to plant carrying the resistance allele, either mock-treated or thrips-exposed.

The second candidate gene is an uncharacterized locus, LOC107874801. This candidate gene did not show significant difference in expression between mock-treated and L1 induced plants that have the resistance allele in homozygous state, but did show a significantly increased expression when plants with the susceptibility allele in homozygous state were induced with L1 larvae. This might indicate that LOC107874801 is a susceptibility gene that plays a role in increasing the compatibility between plant and insect, for instance by blocking the plant’s defence pathway upon induction (Schie and Takken 2014).

The third candidate gene is the predicted gene encoding a protein similar to acid phosphatase 1 (APS1). Acid phosphatase 1 is similar to vegetative storage protein 2 in *Arabidopsis thaliana* (AtVSP), an acid phosphatase which is induced upon wounding, insect feeding, methyl jasmonate and phosphate deprivation (Mason and Mullet 1990; Berger et al. 1995, 2002; McConn et al. 1997; Stotz et al. 2000; Reymond et al. 2004). Due to its acid phosphatase activity, AtVSP causes developmental delays and increased mortality when included in the diet of insects with an acidic gut lumen (Liu et al. 2005). It is hypothesized that thrips have an acidic gut as well, as proteases present in the thrips midgut have an optimum at pH 3.5 (Annadana et al. 2002; Outchkourov et al. 2004). The proteins predicted to result from the susceptibility and resistance alleles of APS1 differ, with two amino acid changes in the susceptibility allele predicted to be deleterious. When the predicted protein change in the susceptibility allele would result in a loss of function of the APS1 protein, it could explain the difference in thrips resistance that we observe when comparing plants with the susceptibility allele in homozygous state, compared to plants with the resistance allele in homozygous state.

As we only studied gene expression at one time point (6 h), we might have missed genes that have differential expression between group R and group S with different treatments at earlier or later time points. For instance, Sarde (2019) showed that many changes in gene expression in pepper upon thrips feeding start 1 h and 2 h after infestation. Therefore, the potential role of other genes located in the QTL region between M12 and M13.4 should not be ignored.

### The role of metabolites in the thrips resistance mechanism controlled by the QTL on chromosome 6

In addition to fine mapping the resistance QTL, we compared plants within one F_4_ line that either had the thrips resistance or susceptibility allele of the fine-mapped QTL region on chromosome 6 in homozygous state, and leaves exposed to first instar (L1) larvae or mock-treated. We performed an untargeted LC–MS analysis of water–methanol extractable metabolites, including capsianosides (diterpene glycosides) previously reported to be associated with thrips resistance, on young leaves of 12 weeks old plants in which the phenotypic effect of the resistance QTL was clearly detectable. No significant differences in the relative abundances of any of the detected metabolites were found between the two contrasting groups of F_4_ plants, neither between L1-exposed and mock-treated samples within each sample group; those few mass peaks that appear to differ significantly between the treatments were most likely artefacts due to yet unexplained differential ionization issues in specific chromatographic regions. This observation suggests that at least the metabolites that could be detected using our extraction and LC–MS method do not play a direct role in the resistance mechanism. We cannot exclude the possibility that specific metabolites do play a role, but at least the 40 diterpene glycosides detectable with our method do not. This finding is not in line with the role in thrips resistance hypothesized for these metabolites in *Capsicum* that was based on association in completely independent studies (Macel et al. 2019; Maharijaya et al. 2019). Also, we expected to observe higher levels of diterpene glycosides upon exposure to thrips, as thrips feeding induces a JA-related defence in plants (Abe et al. 2009; Sarde et al. 2019), and levels of diterpene glycoside increase upon JA-application in *Nicotiana attenuata* (Keinänen et al. 2001; Heiling et al. 2010). From the 40 identified diterpene glycosides, 36 were significantly different between the resistant and susceptible reference plants. One of these putative diterpene glycosides was only present in the resistant reference, but neither in the susceptible reference nor in any of the F_4_ plants. The outcome of this analysis shows that, although we observed differences in putative diterpene glycosides between the resistant and susceptible reference, these diterpene glycosides do not play a key role in the mechanism of resistance controlled by genes located in the QTL on chromosome 6. In addition, we could not detect other metabolites that might play a role in thrips resistance, such as chlorogenic acid, which was more abundant in thrips-resistant compared to thrips-susceptible chrysanthemums (Leiss et al. 2009b).

Twenty-one genes that might play a role in diterpene glycoside synthesis were found in the previously defined QTL region on chromosome 6, as defined by Maharijaya et al. (2015). Fifteen of the 21 genes encode cytochrome P450 (CYP) proteins, which might play a role in the final steps of the diterpene glycoside pathway. As each CYP catalyses a different reaction, it is unlikely that all CYPs play a role in the diterpene glycoside synthesis. Four CYP families, from which seven members were found in the QTL region as defined by Maharijaya et al. (2015), were previously suggested to play a role in terpenoid metabolism (Christoffersen Rolf et al. 1995; Schopfer and Ebel 1998; Ro et al. 2005; Ohnishi et al. 2006; Thornton et al. 2010; Höfer et al. 2013). The CYP89 and CYP94 families, from which eight members were found in the QTL region, do not seem to play a role in the terpenoid pathway (Kahn et al. 2001; Christ et al. 2013). The six other genes encode a geranylgeranyltransferase type-1 subunit alpha, two gibberellin 20 oxidase 1, gibberellin oxidase 3, and gibberellin 3-beta-dioxygenase 1 and 3 enzymes. Geranylgeranyltransferase type-1 subunit alpha is an essential subunit of the geranylgeranyltransferase complex, which plays a role in the transfer of the precursor of diterpenes, i.e. geranylgeranyl-diphosphate, to the cysteine residue of a protein (Yalovsky et al. 1997). Gibberellin 20 oxidase 1 and 3, and gibberellin 3-beta-dioxygenase are involved in the biosynthesis and activation of gibberellin, which belongs to a large family of diterpenoid plant hormones (Williams et al. 1998; Rieu et al. 2008; Zi et al. 2014). However, all these genes, except cytochrome P450 94A2, are located outside of the fine-mapped QTL region conferring thrips resistance. Maharijaya et al. (2019) identified mQTLs that showed overlap with the resistance QTL on chromosome 6, of which two mQTLs were associated with diterpene glycosides. Therefore, it is conceivable that the genes located in these mQTLs play a role in diterpene glycoside synthesis, but that diterpene glycosides themselves do not play a key role in the differential thrips resistance controlled by genes located in the fine-mapped QTL. This suggestion is supported by the metabolomic analysis, as we did observe significant difference in thrips resistance levels between group R and S, but did not detect significant differences in abundance of identified diterpene glycosides nor in any other metabolite detected with our extraction and analysis method.

While it was previously shown that diterpene glycosides are correlated with thrips resistance in *Capsicum* (Macel et al. 2019; Maharijaya et al. 2019), the present results indicate that the putative role of diterpene glycosides in thrips resistance in *Capsicum*, if any, needs to be re-evaluated. The metabolome is the end result of many cellular processes, thus the plant’s ultimate response to genetic and environmental factors. Studying the metabolome is an exploratory tool that needs validation of observed correlations with traits before conclusions about the underlying mechanisms can be drawn (Fiehn 2002; Camacho et al. 2005). Due to the complex connection between metabolites of seemingly unrelated pathways, for instance due to pleiotropic effects, a correlation between metabolites as end products and resistance might not lead to identifying the causal pathway or gene (Fiehn 2002). In order to confirm that diterpene glycosides do not play a role in thrips resistance in *Capsicum*, we suggest to knock-out genes that play a role in diterpene glycoside biosynthesis in resistant *Capsicum* accessions, and determine whether or not this knock-out affected the level of thrips resistance.

## Conclusions

In this study, we validated the previously identified QTL region on chromosome 6 using larval development as a resistance parameter. The QTL region was fine-mapped to 0.4 Mbp. Fifteen candidate genes were identified in this region. By combining the QTL mapping with an RNA-sequencing approach we were able to narrow down the number of candidates, which resulted in the selection of three genes that are likely to play a role in thrips resistance. Further validation of the altered gene expression and predicted protein structures of these three genes is needed to confirm our findings. Also, we conclude that the resistance mechanism underlying this specific fine-mapped QTL most likely works independently from the diterpene glycoside pathway. The selection of candidate genes directs targeted testing of putative mechanisms of resistance in *Capsicum* against thrips.

## Supplementary Information

Below is the link to the Supplementary Information.

Supplementary file 1 (XLSX 177 kb)
